# Minding the Ecological Body: Neuropsychoanalysis and Ecopsychoanalysis

**DOI:** 10.3389/fpsyg.2013.00125

**Published:** 2013-03-25

**Authors:** Joseph Dodds

**Affiliations:** ^1^University of New York in PraguePrague, Czech Republic

**Keywords:** Chaos, complexity, neuropsychoanalysis, ecopsychoanalysis, ecology, climate change, psychoanalysis, Guattari

## Abstract

Neuropsychoanalysis explores experimentally and theoretically the philosophically ancient discussion of the relation of mind and body, and seems well placed to overcome the problem of a “mindless” neuroscience and a “brainless” psychology and psychotherapy, especially when combined with a greater awareness that the body itself, not only the brain, provides the material substrate for the emergent phenomenon we call mind. However, the mind-brain-body is itself situated within a complex ecological world, interacting with other mind-brain-bodies and the “non-human environment.” This occurs both synchronically and diachronically as the organism and its environment (living and non-living) interact in highly complex often non-linear ways. Psychoanalysis can do much to help unmask the anxieties, deficits, conflicts, phantasies, and defenses crucial in understanding the human dimension of the ecological crisis. Yet, psychoanalysis still largely remains not only a “psychology without biology,” which neuropsychoanalysis seeks to remedy, but also a “psychology without ecology.” Ecopsychoanalysis (Dodds, 2011b; Dodds and Jordan, 2012) is a new transdisciplinary approach drawing on a range of fields such as psychoanalysis, psychology, ecology, philosophy, science, complexity theory, esthetics, and the humanities. It attempts to play with what each approach has to offer in the sense of a heterogeneous assemblage of ideas and processes, mirroring the interlocking complexity, chaos, and turbulence of nature itself. By emphasizing the way the mind-brain-body studied by neuropsychoanalysis is embedded in wider social and ecological networks, ecopsychoanalysis can help open up the relevance of neuropsychoanalysis to wider fields of study, including those who are concerned with what Wilson (2003) called “the future of life.”

## Embedding the Mind-Body in Ecological Space

We are taking part in a planetary pyramid scheme, getting into an ecological debt from which there can be no bail outs. We live on a finite planet but have an economic system predicated on unending growth. Scientists estimate human demand already exceeded the biosphere’s regenerative capacity in the 1980s (Wackernagel et al., 2002, 926), yet somehow this just doesn’t hit home, our behavior doesn’t match our knowledge. Why? This paper draws on ideas from my recent book *Psychoanalysis and Ecology at the Edge of Chaos* (Dodds, 2011a) to explore the possibility of a non-linear ecopsychoanalysis (Dodds, 2011b) with which to respond to a climate at the edge of chaos.

**Figure 1:**
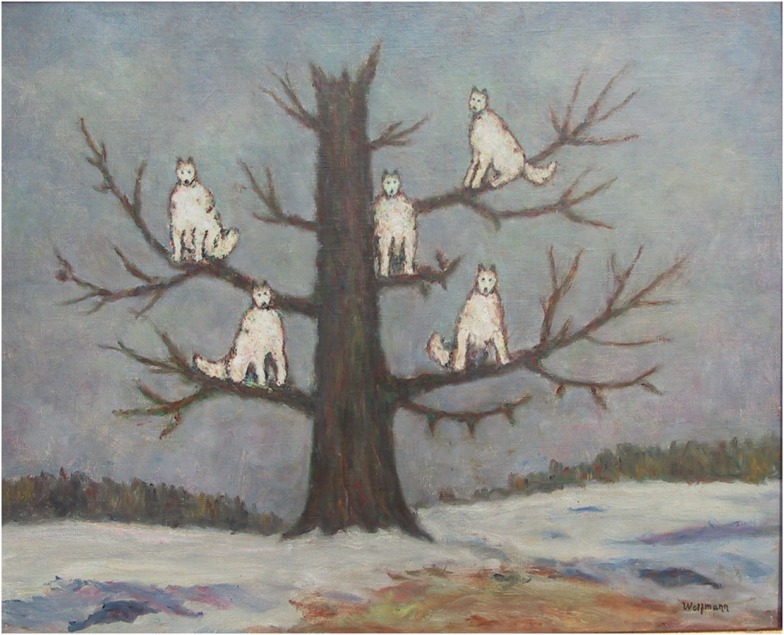
**Painting by the “wolfman” (Segei Pankejeff 1886–1979), from Freud (1918, 30)**.

What do we see when we look at this famous painting by the “wolfman” (Figure 1)? Well we know what Freud (1918) saw: Daddy, penis, castration. He didn’t really see the wolves, the other than human world. The wolves could only be stand-ins for the totemic father, symbolic of human, all-too-human relationships. Genosko (1993) and Deleuze and Guattari (2003a), point out that Freud’s analysis enacts a reversal of the stare. From the dream where the wolves are watching, intensely watching, the sleeping child, it is now the human child watching the primal scene of his parents copulating. He thus enacts a domestication of the animal gaze, territorializing it into the affective economy of Oedipus, thereby embodying aspects of our civilization’s relation to non-human world. But what if we refuse this reversal? What happens if we reflect on the terror and fascinating in the gaze of the wolves: their “conversation of death” (Lopez, 1978)? What do *the wolves* see when they look at us?

A key problem identified by ecopsychology is how to overcome our experiential, academic, and scientific separation from nature. In so far as the division of mind and body is central for this divide, neuropsychoanalysis deserves to have a place in this developing research. Neuropsychoanalysis explores experimentally and theoretically the philosophically ancient discussion of the relation of mind and body, an issue at the heart of psychoanalysis from the start, with its early work on hysterical conversion (Breuer and Freud, 1895; Freud, 1905a) and later developments in the psychosomatics of the Paris School and beyond (Marty et al., 1963; Aiesenstein and Rappoport de Aesemberg, 2010). Neuropsychoanalysis seems well placed to overcome the problem of a “mindless” neuroscience and a “brainless” psychology and psychotherapy, especially when combined with a greater awareness that the body itself, not only the brain, provides the material substrate for the emergent phenomenon we call mind (Stora, 2007). However, there is a further term missed from this perspective, which has more relevance now than ever, which is the fact that the mind-brain-body is itself situated within a complex ecological world, interacting with other mind-brain-bodies and with all that psychoanalyst Searles (1960, 1972) referred to as the “non-human environment.” This occurs both synchronically (webs of interactions at a given moment in time) and diachronically (e.g., the interactions over evolutionary deep time), as the organism and its environment (living and non-living) interact in highly complex often non-linear ways.

Ecopsychology has argued that the split of mind from its wider ecological matrix is as disastrous as the related Cartesian split between mind and body, and is reflected in the current environmental crises we face (Roszak et al., 1995; Buzzell and Chalquist, 2009). However, ecopsychology has problems of its own, in particular its tendency toward an “eco-mysticism” that a more engaged relationship with the sciences of ecology, evolutionary biology, and neuroscience (cognitive, social, affective, and developmental) can help to counteract. The idea of the ecological body is a development of ecopsychology’s suggestion of an “ecological self” and an “ecological unconscious,” with ecological relations and attachments seen as developing alongside more traditionally conceived object relations (Jordan, 2009).

In our era of anxiety, denial, paranoia, apathy, guilt, and despair in the face of climate change, there is an urgent need for a critical dialog between psychoanalysis and ecology, for minding the ecological body. Psychoanalysis can do much to help unmask the anxieties, deficits, conflicts, phantasies, and defenses crucial in understanding the human dimension of the ecological crisis (Bigda-Peyton, 2004; Randall, 2005; Morton, 2007; Žižek, 2007; Bodnar, 2008; Rust, 2008). Yet, psychoanalysis still largely remains not only a “psychology without biology,” which neuropsychoanalysis seeks to remedy, but also a “psychology without ecology.” This is the role ecopsychoanalysis seeks to fulfill. While it includes applications of psychoanalysis to ecological problems it seeks in addition to apply ecological understandings and approaches to psychoanalysis. In Dodds (2011a) I suggest there is a need for a meta-perspective to help integrate the various levels (psychological, social, biological, neurological, cultural, and ecological). The philosophy of Deleuze and Guattari (2000, 2003a,b) combined with the sciences of complexity (Bonabeau et al., 1999; Sole and Goodwin, 2000; Piers et al., 2007) and the systems theory of Gregory Bateson (2000, 2002), help create a framework for integrating the diverse levels of analysis required in any comprehensive attempt to deal with the ecological crisis we now face.

Neuropsychoanalysis not only provides a more sophisticated and integrated understanding of the mind-brain (including recent advances from social neuroscience) necessary for any attempt to deal with the psychology of the environmental crisis, it also offers a model for how such a difficult interdisciplinary project might work. In addition, by emphasizing the way the mind-brain-body studied by neuropsychoanalysis is embedded in wider social and ecological networks, ecopsychoanalysis can help open up the relevance of neuropsychoanalysis to wider fields of study, including those who are concerned with what Wilson (2003) called “the future of life.” Ecopsychoanalysis (Dodds and Jordan, 2012) is a new transdisciplinary approach to thinking about the relationship between psychoanalysis, ecology, “the natural,” and the problem of climate change. It draws on a range of fields including, psychoanalysis, psychology, ecology, philosophy, science, complexity theory, esthetics, and the humanities. It attempts to play with what each approach has to offer in the sense of a heterogeneous assemblage of ideas and processes, mirroring the interlocking complexity, chaos, and turbulence of nature itself.

## Dreaming at the Precipice

The climate crisis is also a crisis of theory. Academia has divided human thought into a schizoid fragmented space but climate change forces us to think transversally, about a world of unpredictable, multiple-level, highly complex, non-linear interlocking systems. There is therefore a need for a way of thinking able to integrate the disparate strands of analysis, related to what Bion (1984) calls the work of linking, connected with the alpha-function and the dreamwork. Bion describes building links between mental objects, and the attack on linking characteristic of psychosis. When “alpha-function” is compromised we are left with undigested fragments of experience: “beta-elements” incapable of being woven into the tapestry of our psychic landscapes. We require a means of linking diverse elements together without losing their specificity.

**Figure 2:**
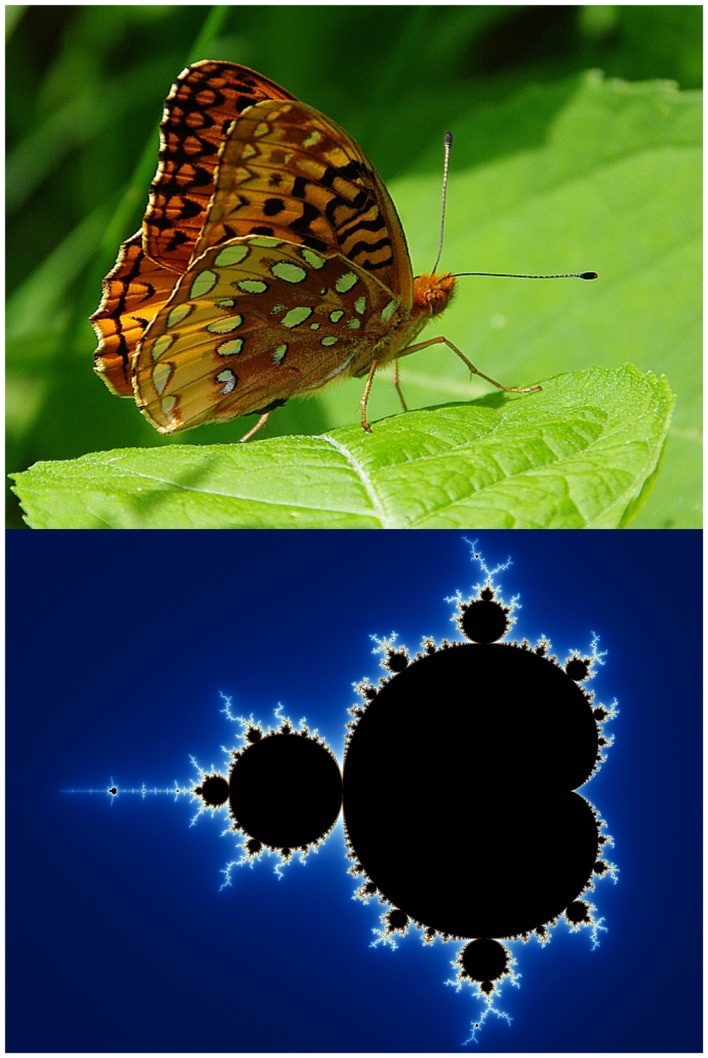
**Complexity as meta-theory**.

Here I turn to the non-linear sciences of complexity and chaos (Figure 2), and the philosophy of Deleuze and Guattari. In his book *Chaosmosis*, Guattari (1995, 91) called for a generalized science of ecosystems or “ecosophy,” a generalized machinics with “resonances, alliances, and feedback loops between various regimes, signifying and non-signifying, human and non-human, natural and cultural, material and representational.” In Dodds (2011a), I argue there is as much a need to bring non-linear and ecological thinking into psychoanalysis as for a psychoanalytic approach to ecology, taking seriously the possibility of thinking in terms of what Guattari (2000) called in his final book, *The Three Ecologies*, the ecologies of mind, nature, and society.

Science has developed with the concept of linear systems but is increasingly realizing these represent a special case in an otherwise non-linear world. We can think about this in terms of *attractors*, points toward which a system tends to converge. Any variation in starting point within the *basin of attraction* is canceled out by the powerful pull of the attractor. Within limits as our global temperatures increases, *negative feedback* processes act to draw the system back to a more central point, the *point attractor* (other more complex attractors include “periodic” attractors and “strange” or “chaotic” attractors). When moved toward the edge of the basin of attraction we reach *bifurcation* points, where non-linearities rule as the slightest difference in starting conditions or tiniest fluctuation causes a radical shift, a *phase transition* to a new attractor or set of attractors.

Scientists suggest our climate may be approaching several such tipping points, or perhaps it has already begun, with potentially lethal *positive feedback* processes no longer capable of being damped out. A non-linear perspective is crucial not only for climate science (Sawaya, 2010) but for the psychology of climate change. Our familiar ways of thinking assume a linear relationship between CO_2_ emissions and a warming, that there will always be time later to turn it around. This is a failure in our mental ecology which leads, via pathological forms of social ecology, into potential catastrophic collapses of natural ecology.

## Psychoanalysis and Climate Change

Before exploring further the meshwork (DeLanda, 2005) for integrating the three ecologies we need to begin with the ecology of mind, and especially the ecology of affect, and thus, despite all its faults, the territory of psychoanalysis. Freud (1927) claimed civilization arose to defend us against nature, but that the aim of achieving total control over either our inner nature or the outer world was a dangerous illusion to defend against feelings of helplessness and fear in the face of the awesome power of mother nature, of acknowledging dependency on this largest of “holding environments,” the ultimate “environment mother” (Winnicott, 1987).

Freud’s “eternal adversaries,” Eros, and Thanatos, are unfortunately unlikely partners in their destructive effects on nature. The “nirvana principle’s” desire for non-existence/annihilation can be seen in our virtual indifference toward the world’s sixth great mass extinction, and in the apocalyptic rhetoric of the environmental movement and recent “eco-disaster” films. For Žižek (2007) “The world without us” is…fantasy at its purest: witnessing the Earth itself retaining its pre-castrated state of innocence, before we humans spoiled it with our hubris.” Eros, through over-consumption and overpopulation, also works toward the potential collapse of the biosphere (Bigda-Peyton, 2004). However, in the form of “biophilia” (Wilson, 2003) Eros can work to reinvigorate our love of nature which may help us turn back from the brink.

To explore climate denial further, we can turn to a joke Freud (1905b, 62) used to illustrate the logic of the unconscious (Freud, 1911; Matte-Blanco, 1998). When a man is told he should replace a pot he borrowed and returned damaged, he refuses, claiming: (1) I returned it undamaged. (2) The hole was there when you gave it to me. (3) I never borrowed it! These mutually contradictory answers alert us to unconscious processes united by the motivation to remove the blame and prevent need for action, and correspond well with arguments against action on climate change.

1. *There’s nothing wrong with the climate kettle*: (here climate change is seen paranoiacally as a conspiracy by UN/communists/Al Gore to destroy our freedom or instead of capitalists trying to stop poor countries developing.) Alternatively, “the evidence is not conclusive” (the IPCC’s “unequivocal” is not unequivocal enough). While this may appear more rational on the surface, in its own term it involves playing Russian Roulette with the entire planet.
2. *There was a hole in the planet when you gave it to me*. (not caused by humans, or caused by other humans, either way, not-me, not my problem). However, unconscious deflection of guilt does nothing to stop the disastrous consequences of climate change so we would still need to take urgent action. One psychoanalytically interesting conclusion is at times people can fear guilt more than their own, or everyone’s, destruction.
3. *There is nothing we can do about it*, also found in burnt out environmentalists filled with feelings of despair and disempowerment, which Jungian ecopsychologist Rust (2008) calls the “we’re completely fucked” defense, allowing us to give up thinking.

The different arguments relate to defenses against specific anxieties. *Its not happening* involves more psychotic defenses against paranoid-schizoid anxiety (extinction, annihilation). *Its not our/my fault* involves neurotic defenses against depressive anxiety (difficulty in acknowledging human culpability and guilt). *There’s nothing I/we can do about it* is closest to recognizing the problem but without realistic reparative possibilities the individual is stuck with the despair and pain of the depressive position without hope. As Searles (1972, 366) put it, “instead of feeling isolated within emotional depression, one feels at one with everyone… in a “realistically” doomed world.” Such defenses need to be understood not only individually, but as involving unconscious alliances (Kaes, 2007) created socially, through small interactions at all levels giving rise to *social phantasy systems* (Jaques, 1955). In complexity theory which Palombo (2007) suggests is a suitable ‘parent science’ for psychoanalysis this is an example of *self-organization* (SO), where lower levels interact to form higher level structures embodying emergent properties which then feedback to lower levels in a process of ongoing recursivity.

## Object Relations and Ecological Relations

**Figure 3:**
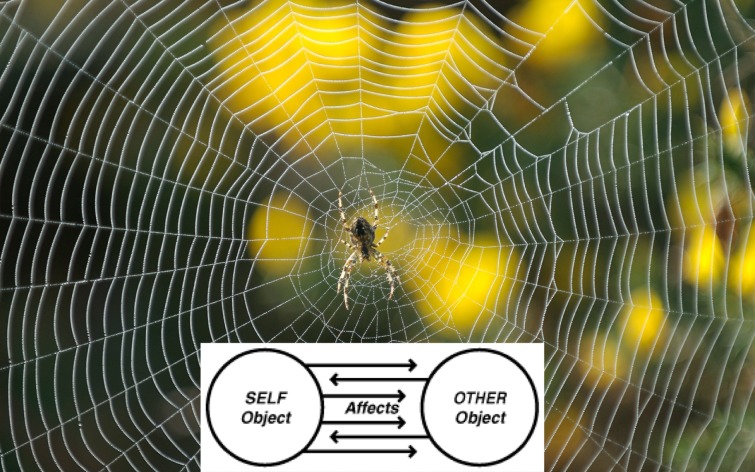
**Object relations and ecological relations**.

Object relations, emphasizing the self as constituted in and through relational webs, moves psychoanalysis in an ecological direction (Figure 3). Development involves moving from “absolute dependence” to “mature dependence” (Fairbairn, 1992), suggesting a vision for a more mature culture, with self and society seen as inextricable from its relations to other beings, to ecological webs, and to the Earth. For Searles (1972, 368) “an ecologically healthy relatedness to our non-human environment is essential to the development and maintenance of our sense of being human” which has become “so undermined, disrupted, and distorted, concomitant with the ecological deterioration, that it is inordinately difficult for us to integrate the feeling experiences, including the losses, inescapable to any full-fledged human living.”

Traditionally, psychoanalysts would analyze environmental concern as reflecting “deeper” feelings relating to human “objects,” but human conflict could equally be a displacement from anxiety concerning the environment. If we broaden Winnicott’s “holding environment” to include the holding environment of the Earth, we can understand how realizing the enormity of the crisis can threaten psychological disintegration and collapse. Furthermore, not only is environmentally damaging behavior a form of addiction (e.g., consumer items functioning as Kohutian selfobjects to shore up a fragile self, Kohut, 1985; Winnicott, 1987, 1999), but addictions can also arise to deal with anxiety concerning our damaged world as discussed by Susan Bodnar (2008). Psychoanalysts need to recognize engagement with ecology is not only for “applied” psychoanalysis, but is crucial to its core clinical domain.

The phrase “Mother Earth” suggests our experience with the planet relates to our experience with our (m)other. Not only feelings of love and being held, but phantasies of an infinitely giving Earth-breast we feel entitled to suck on with ever increasing intensity without limit. Unable to tolerate weaning, our response to ecological crisis includes rage, envy, and destructiveness, including spoiling and omnipotent attacks on the earth-breast. Meltzer’s (1994) “toilet-breast” concept is useful here. Psychologically the breast is not only a provider of nutrition, but a place where we expel unbearable states of mind.

Various developmental levels may intersect with our problematic relationship with nature. The apocalyptic threat of climate change may evoke the extremely primitive persecutory anxieties of Klein’s paranoid-schizoid position, leading to omnipotent defenses to protect against feelings of helpless and fragmentation (Jordan, 2009). The paranoia surrounding climate change allows the “bad sadistic enemy” to be fought against “not in the solitary isolation of the unconscious inner world, but in co-operation with comrades-in-arms in real life” (Jaques, 1955, 483). Searles (1972) points out that ironically there is now a certain objectivity to schizophrenic “end of the world” fantasies. This can lead many to intuitively feel ecological warnings are “crazy” and we shouldn’t listen to them, partly out of fear of contamination because they touch a “crazy” part of all of us.

At the phallic-Oedipal level, Searles (1972, 364) identifies phantasies of eliminating Oedipal rivals (inc. future generations) and the “moralistic” tone of much ecological writing involving projecting Oedipal guilt, accusing us of raping mother earth. In addition, through relentless advertising, possessions such as cars have become symbols of (male) genital achievement and initiatives to reduce car use can feel like castration (Randall, 2005). The ecological version of Klein’s (1987) depressive position involves mourning for environmental destruction, guilt for the damage done, a growing awareness the lifestyles and civilization we are so proud of are causing such damage to planetary ecosystems, and a reparative drive to restore, repair, and recreate the lost and damaged world (internal and external).

The environmental crisis forces us to face the traumatic aspects of transience, that nothing is permanent. Drawing on Freud’s (1916) concept of anticipatory mourning, we might expect individuals and societies to adopt positions of consciously not caring about the environment or even our species survival, or becoming actively destructive and self-destructive, as a defense against the mourning yet to come. Alternatively we may engage in a premature anticipatory mourning, falling into a despair preventing the very action which might avoid the feared loss, while there is still time. Freud (1916, 306) urges us to face with honesty and courage the fact that: “[a] time may indeed come when the pictures and statues which we admire to-day will crumble to dust, or a race of men may follow us who no longer understand the works of our poets and thinkers, or a geological epoch may even arrive when all animate life upon the earth ceases.” In the face of the enormous pain and fear the ecological crisis evokes, we need to find effective means of reparation, to restore, and recreate the damaged world, inside and out. Without hope that meaningful, as opposed to manic, reparation is possible, we have only the choice between denial, madness, and despair. As psychoanalysis opens itself up to a greater awareness of the web of life, the object-related self and the narcissistic self needs to be viewed as developing alongside the ecological self.

## Biophilia and Biophobia

**Figure 4:**
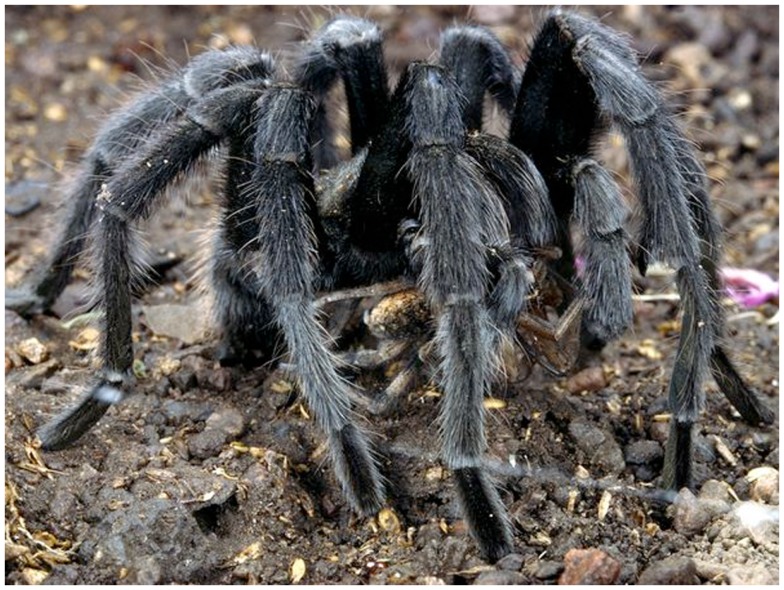
**Biophila and biophobia**.

While ecopsychology in its classic form is in danger of creating a new mysticism or a new religion, there is much of value within the tradition so we shall see what symbioses can occur in this ecology of ideas. Where Freud saw the oceanic feeling as “something like the restoration of the limitless narcissism,” Roszak et al. (1995, 12) instead sees it as reclaiming the repressed of the ecological unconscious. This is connected to what the zoologist Wilson calls “biophilia” “the innately emotional affiliation of human beings to other living organisms” (Kellert and Wilson, 1993) (Figure 4), a consequence of our long evolution and adaptation to the natural world and for Wilson (2003) a crucial force in countering the biodiversity crisis.

Wilson’s actual claim is less global than his ecopsychological readers often assume. In particular, the innate preference we seem to have for natural environments refers specifically to certain *kinds* of natural environments, the ecology our species evolved to live within, predominantly savanna environments and transitional woodland and it is therefore ultimately an argument underpinned by evolutionary theory, and one which for Wilson (2003, 137) connects esthetics to biology via the pleasures arising from our evolved brain.

However, Wilson (2003, 137) emphasizes we should not understand this as hardwiring, but rather that we are predisposed to find certain environments preferable to others: “Psychologists who study mental development say that we are hereditarily *prepared* to learn certain behaviors and *counter prepared* to learn others.” For example, the “vast majority of humans… are prepared to learn the lyrics of a song but counter prepared to learn calculus.” Crucially for psychoanalytic and ecopsychological perspectives, such preparedness usually involves “sensitive periods during childhood and early maturity in which learning and distaste are most easily picked up.”

Thus, Wilson’s biophilia is something that can be learned, encouraged, and developed. It refers not to a fixed instinct but an innate tendency toward a connection with the natural world which can be nurtured or not, especially during the crucial stages of child development of such interest to psychoanalysts. He goes on to describe the stages of the acquisition of biophilia which can be interestingly compared to Freud’s work on children’s relations with animals (Genosko, 1993). Although Wilson does not say this, in some ways we could describe our culture as remaining stuck within the first stage of the development of biophilia.

The critical states in the acquiring of biophilia have been worked out by psychologists during studies of childhood mental development. Under the age of six, children tend to be egocentric, self-serving, and domineering in their responses to animals and nature. They are also most prone to be uncaring or fearful of the natural world and of all but a few familiar animals. Between six and nine, children become interested in wild creatures for the first time, and aware that animals can suffer pain and distress. From 9 to 12 their knowledge and interest in the natural world rises sharply, and between 13 and 17 they readily acquire moral feeling toward animal welfare and species conservation (Wilson, 2003, 137–138).

The timing of these stages obviously show a fairly large variability among individuals. It is also worth exploring the developmental stages of children’s relation to the environment as a whole. This research supports other findings we shall study below, when we will review the significant health benefits (psychological and physical) of living near green spaces, for children and adults. Studies show that children tend to move from confining themselves to the immediate vicinity of their home and the small creatures found there (around age four) to exploring “nearby woods, fields, ditches, and other unclaimed spots they can claim as their own” (Wilson, 2003, 138) between approximately the ages of 8 and 11.

As Wilson (2003:, 138) writes, drawing on Sobel’s (2001) book *Children’s Special Places*, here they “often build some kind of shelter such as a tree house, fort, or cave where they can read magazines, eat lunch, conspire with a friend… play games, and spy on the world.” If natural environments are not available, as for example in areas such as East Harlem, children will instead build forts “in culverts, alleyways, basements, abandoned warehouses, railroad right-of-ways, and hedges.” The secret places of childhood, connect us to place, and help in our psychological development. Importantly for Sobel, “if played out in natural environments, they also bring us close to the earth and nature in ways that can engender a lifelong love of both.”

However, if we accept that biophilia is an innate tendency in human nature, we must also accept the possibility, or even the likelihood, that “biophobia” is just as natural. This is a subject that ecopsychologists are often conspicuously absent in addressing. This deep acceptance of the ambiguity of our relationship with nature found in Wilson, is something perhaps Freud would have appreciated. As Wilson (2003, 141) writes, throughout “most of human deep history there have been predators eager to snatch us for dinner; venomous snakes ready with a fatal, defensive strike to the ankle; spiders and insects that bite, sting, and infect; and microbes designed to reduce the human body to malodorous catabolic chemicals…the reverse side of nature’s green-and-gold is the black-and-scarlet of disease and death.” In a similar way to biophilia, our biophobic tendencies can be encouraged and further developed, or reduced and alleviated, through critical developmental experience.

At one end of the scale are mild distaste and feelings of apprehension. At the other end are full-blown clinical phobias that fire the sympathetic nervous system and produce panic, nausea, and cold sweat. The innate biophobic intensities are most readily evoked by sources of peril that have existed in the natural world throughout humanity’s evolutionary past. They include heights, close spaces, running water, snakes, wolves, rats and mice, bats, spiders, and blood. (Wilson, 2003, 141).

Such prepared learning is generally not found for contemporary threats which are objectively far more dangerous such as frayed wires, guns, or cars. In ways compatible with psychoanalysis, the expression of such fears can occur unconsciously. For example when psychologists flash images of spiders or snakes subliminally (15–30 ms), subjects already conditioned negatively to these particular natural “threats” react physically (for example by facial muscle changes) without consciously registering the experience at all (Wilson, 2003, 142).

Biophilia and biophobia can be understood as the ecopsychological equivalent of Freud’s (1920), Eros, and Thanatos. For more research on the development of biophobia and biophilia, see for example Kellert and Wilson (1993), Orr (1994), Wilson (2003). In Dodds (2011a) I also explore biophobia from the point of view of an ecopsychologically sensitive psychoanalytic and schizoanalytic approach to the becoming-animal theme in horror films (Creed, 2005; Powell, 2006) and its clinical manifestation in the form of animal phobias (Freud, 1909).

## Ecopsychology and Health

Ecopsychologists have been interested in studying the psychological impacts of life in an age of ecological crisis. Heinberg (2009, 198) suggests that in this context we need also to consider the idea of *eco-grief*, the feelings of loss connected to ecological devastation and the threatened loss of a whole way of life, which one way or another, is about to come to an end, in what he calls *pre-traumatic stress disorder*, related in many ways to Freud’s (1916) anticipatory mourning. He suggests a psychological approach using the stages of grief described in the well-known Kübler-Ross (1973, 2005) model (denial, anger, bargaining, depression, acceptance) to understand where we are as a society and as individuals. From this perspective, different types of interventions might be more or less “effective for helping people accept our situation, depending on their current stage of adjustment” (Heinberg, 2009, 198). He suggests, however, that the classic stages are not enough, because beyond acceptance there needs to be action, not only due to the ecological urgency, but because accepting “the reality all too often leads to depression and despair.”

**Figure 5:**
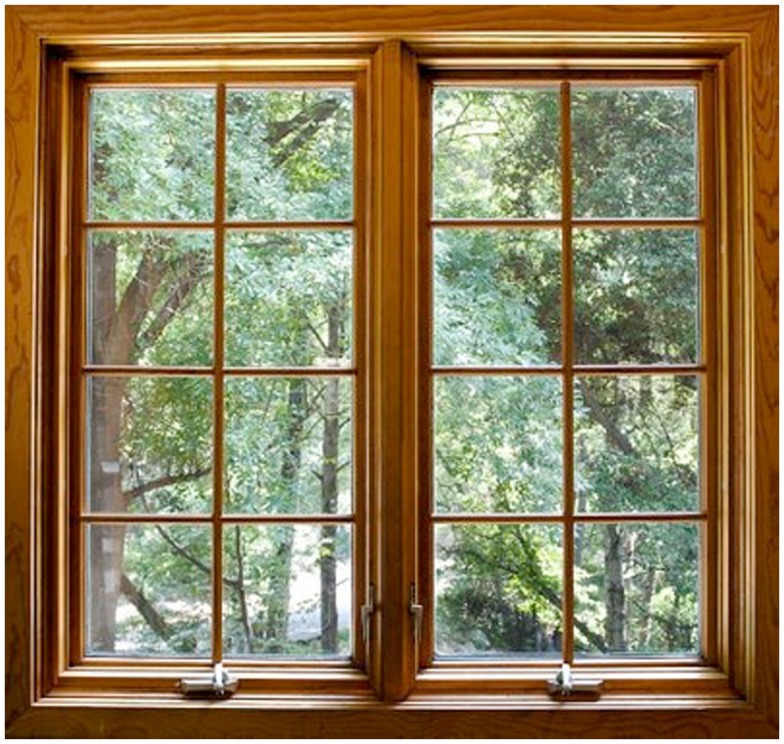
**Physical and psychological health and natural spaces**.

Although Santostefano (2008) cautions us against a naïve version of ecopsychology that assumes nature automatically generates a sense of well-being and improvements in physical and mental health, there does seem to be an increasing amount of empirical evidence to support the contention that nature heals (e.g., Mind, 2007; Chalquist, 2009) (Figure 5). Researchers from the VU University Medical Center in Amsterdam recently conducted a large study of 3,50,000 people showing that living near green spaces had substantial physical and mental health benefits (British Broadcasting Corporation, 2009). The greatest benefits were for those living less than a kilometer away and the largest positive impacts were on anxiety disorders and depression. Living near green areas reduced depression rates by 21% for children under 12. Physical disorders, such as heart disease, diabetes, stomach and respiratory infections, and neck, shoulder, back, wrist and hand complaints, also showed substantial improvements. In addition, research by Ulrich (1984) has shown that the view from a hospital window (whether natural or concrete) has a significant and measurable effect on the speed and completeness of a patients recovery (Ulrich, 1984; Verderber and Reuman, 1987).

Further evidence supports the therapeutic effect of nature and thus further confirms Wilson’s (2003) biophilia hypothesis. Wilson (2003, 139–140) summarizes some of these findings:

120 volunteers were shown a stressful movie, followed by videotapes of either natural or urban settings…they recovered from the feeling of stress more quickly while experiencing the natural settings…supported by four standard physiological measures of stress: heartbeat, systolic blood pressure, facial muscle tension, and electrical skin conductance… Studies of response prior to surgery and dental work have consistently revealed a significant reduction of stress in the presence of plants and aquaria… Postsurgical patients recover more quickly, suffer fewer minor complications, and need smaller dosages of painkillers if given a window view of open terrain or waterscape. In one Swedish study covering fifteen years…clinically anxious psychiatric patients responded positively to wall pictures of natural environments, but negatively…to most other decorations… Comparable studies in prisons revealed that inmates provided window views of nearby farmlands and forests, as opposed to prison yards, reported fewer stress-related symptoms such as headaches and indigestion.

Pets can also have a major positive impact on our health, as independent research in Australia, England, and the United States has shown. Wilson (2003, 140) writes that “one Australian study, which factored out variation in exercise levels, diet, and social class, pet ownership accounted for a statistically significant reduction of cholesterol, triglycerides, and systolic blood pressure” while in a similar US study “survivors of heart attacks…who owned dogs had a survival rate six times higher than those who did not” (Wilson, 2003, 140). Unfortunately, cat ownership did not provide the same positive effects. For more information on the health effects of pets and natural environments, see Ulrich (1991, 1999, 2000), Ulrich et al. (1991), Kellert and Wilson (1993), Frumkin (2001) and Frumkin and Louv (2007).

Following a review of this increasingly impressive body of evidence, the mental health charity Mind (2007) strongly supported the benefits of “ecotherapy” and called for the “greening” of mental health provision. This has lead a number of therapists, including those coming from traditional psychoanalytic backgrounds, to explore the possibility of “ecotherapy,” which covers a wide variety of approaches, including taking psychotherapy outside the traditional consulting room into the outdoors (Buzzell, 2009). Jodran and Marshall (2010, 345) explore the various complex clinical factors involved in such a shift, in particular focusing on its impact on boundaries and the therapeutic frame (as both emotional and geographical space) from a relational perspective. Here, the “relational encounter within the dynamic nature of the natural world can provide rich opportunities for a new experiencing with immediacy for both therapist and client, all of which can be fed in to the therapeutic process” (Jodran and Marshall, 2010, 349). Moving outdoors may also enhance mutuality (not identical with equality), given that the space within which therapy occurs is not owned by the therapist, and the process of choosing different terrain can become more a co-created ongoing experience within the therapeutic relationship. Placing therapy outdoors results in the normal static “backdrop” of therapy becoming “a living presence…(where) therapist and client are constantly aware of (both consciously and unconsciously), and responding to, the presence of this vibrant living third in the dynamic” (Jodran and Marshall, 2010, 353–354).

## Dark Ecology: Ecology Without Nature?

In contrast with the call for reconnection at the heart of ecopsychological theory and practice, Morton’s (2007) plea for an “ecology without nature” uses ecocriticism to deconstruct the ecological imaginary, helping us become more aware of how we use “nature” psychologically in ways which get in the way of genuine environmental practice. For Žižek our very idea of “Nature” is a problem: “there is no big Other (self-contained symbolic order as the ultimate guarantee of Meaning); there is also no Nature *qua* balanced order of self-reproduction whose homeostasis is disturbed…by imbalanced human interventions…what we need is ecology without nature: the ultimate obstacle to protecting nature is the very notion of nature we rely on.” An example of the “end of nature” is Morton’s (2010) claim that the “weather conversation” no longer functions. In our era of global warming, weather (as background) no longer exists, it now becomes a mere cipher for that threatening hyper-object we call “climate.” For Morton, without background the foreground also disappears, and rather than retreating into comforting fantasies of “Hobbit-like” Heideggerian “life-worlds,” he encourages us to embrace *dark ecology* involving a “melancholic ethics.”

However, there is a danger ecocritique can remove a primary motivation of the environmental movement. Discourses of “nature no longer existing” may feed into psychological defenses by arguing that as “nature” is already so altered by human activity that “wilderness” doesn’t really exist, there is no reason to protect a nature which has no substance.” In addition, this approach can lose sight of the fact that the ecological crisis ultimately reaches beyond any linguistic constructions, and is not itself a “text” which can be “deconstructed,” but a “Real” beyond language, traumatically rupturing the Symbolic order. Deconstructive approaches also have difficulty in giving ontological space to nature and the material as anything other than an effect of language, or its negation as the “Real.”

With their mixed semiotics, Herzogenrath (2008, 3) claims a Deleuzo–Guattarian ecology “allows for the incorporation of the workings of the ‘repressed’ of representation…of the ‘real’, of ‘nature’ ”. According to Bonta and Protevi (2004, 4), Deleuze and Guattari’s engagement with complexity theory “helps break free of the postmodernist trap by rethinking sense and reference,” shattering “postmodernist equations of signs with signifiers,” such that “at critical thresholds…physical and biological systems can be said to ‘ense’ the differences in their environment that trigger self-organizing processes.” A non-linear reading of Deleuze and Guattari offers not with a flight into eco-mysticism, or a naïve positivist reductionism, or even a postmodernist play of signifiers, but an “intelligent materialism,” a “geophilosophy.”

## Complexity Theory and Self-Organization

[W]hat would thinking be if it did not constantly confront chaos?… chaos has three daughters…the Chaoids – art, science, and philosophy… [Each] cut through the chaos in different ways. The brain is the junction – not the unity – of the three planes (Deleuze and Guattari, 2003b, 208).

Complexity and chaos theories have strong implications for psychoanalysis (Palombo, 2007; Piers et al., 2007) and have “changed the basic concept of the human mind itself” (Guastello, 2004, 4), providing a new way of thinking about the three ecologies. Concepts such as “phase space” embody complex relationships and dynamic processes of change, providing what Deleuze and Guattari call an “abstract machine,” embodying a structural pattern of relationships in many separate heterogeneous domains. For Deleuze and Guattari (2003a, 514), “every abstract machine is linked to other abstract machines, not only because they are inseparably political, economic, scientific, artistic, ecological, cosmic – perceptive, affective, active, thinking, physical, and semiotic – but because their various types are as intertwined as their operations are convergent.”

**Figure 6:**
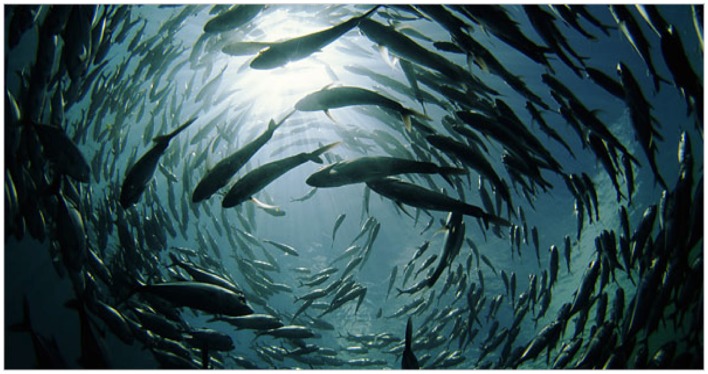
**Self-organization and swarm intelligence**.

*Self-Organization*, deriving partly from for example studies of social insects, occurs when global patterns *emerge* from interactions among lower level components rather than being imposed from outside the system, or any type of “leader,” the “solution without a General” in Deleuze and Guattari’s terms. For Palombo (1999, 24) SO is “the most significant missing ingredient in psychoanalytic theory,” showing how small pieces of insight self-organize into ever larger structures. As Sole and Goodwin (2000, 18) write “Self-organizing behavior emerges unpredictably in systems at different levels” with emergent properties which “provide the recognition that nature can be creative while denying the occurrence of miracles.” The functions as an “abstract machine,” (Deleuze and Guattari, 2003a) embodying a structural pattern of relationships occurring in many separate registers, including the psychological, ecological, and social of Guattari’s (2000) three ecologies. Supporting Deleuze and Guattari’s contention that every individual is fundamentally a pack, the mind/brain itself is increasingly being studied through the virtual diagram of the swarm (Figure 6).

[L]ike the brain, the colony is formed by many individuals in interaction; individual units can switch from one type of activity to others; they can fail or be removed without any harm to the collective…the main differences arise from connectivity: direct contact between individual ants is a transient phenomenon, whereas synaptic connections among neurons usually have a long lifetime… This is partially compensated (particularly in large colonies) by the use of chemicals, which can create spatial structures that clearly involve long-term memory effects (Sole and Goodwin, 2000, 148–149).

Gordon (1999) suggests a similar pattern can be found behind “molecular interactions within a living cell, the unfolding pattern of cells and tissues in an embryo, and the activity of the neurons that produce the mind.” Any complex system can be viewed as a *morphogenetic cascade*, which can include flows from all registers. Thus we can see how the scientific apparatus of complexity theory, along with the philosophical perspective of Deleuze and Guattari can help to provide a meta-perspective from which to connect the various levels of mind, brain, society, ecology, and climate, which this book argues is necessary to allow joined-up-thinking on the topic of climate change and the psychological dimensions of the ecological crisis.

## Life at The Edge of Chaos

**Figure 7:**
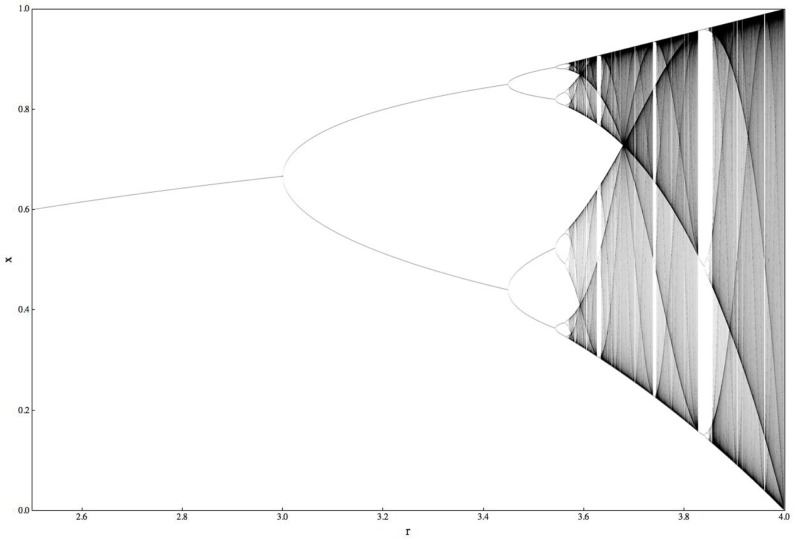
**The march to chaos (Camazine et al., 2001)**.

Climate change is sometimes referred to as “climate chaos” because of the increasingly unpredictable nature of natural systems. A non-linear perspective is thus crucial for climate science, but it also provides ways of engaging with the crisis on the social and psychological levels. *Chaos theory* shows us paradoxically, that chaos is far from the opposite of order and structure. Chaos is a feature of all non-linear systems, which show us that traditional linear approaches to scientific analysis only describe a special case situation within a larger non-linear world. As Deleuze and Guattari (2003b, 119) write, “Science is haunted not by its own unity but…by all the limits or borders through which it confronts chaos.” We can see this relation between chaos and non-linearity in the ‘period doubling’ route to chaos, seen in a wide range of systems describing everything from population growth to fluid dynamics. In Figure 7, we can see that as the growth parameter ‘r’ is increased we move from a ‘point attractor’ (one steady state the system tends to converge on whatever the starting point) to periodic attractors (period 2, then 4, then 8, etc.) with each subsequent ‘bifurcation’, coming increasingly rapidly, until the whole system moves beyond the periodic attractor to the ‘strange’ or chaotic attractor (in the right hand region of the picture). According to Camazine et al. (2001, 43):

As r [the growth rate parameter] is increased…the system not only fails to reach a stable value but also does not oscillate among a number of fixed values. Instead, no pattern occurs… The system is said to be chaotic… [involving a “strange” or chaotic attractor]… Deterministic chaos is the unpredictable behavior of a non-linear system within a certain parameter range… What is so unexpected, however, is that a deterministic equation can yield unpredictable results.

Chaos is essential for SO as the latter involves the amplification through positive feedback of fluctuations created by phenomena such as “random walks, errors, random task-switching” (Bonabeau et al., 1999, 10). The fact that ants regularly get lost used to puzzle scientists who wondered why this “inefficiency” wasn’t eliminated by evolution, but lost foragers can sometimes find new food sources and therefore randomness enhances the creativity of a system or what Bateson (2000) calls its *ecological flexibility*. This is true in psychological, social, biological, and even non-living systems such as swarm robotics.

Similarly, and counter-intuitively, studies of electroencephalograms (EEG’s), electrocardiograms (ECG’s), and other biorhythmic measurements, show *healthy* rhythms have greater turbulence or irregularity (complexity) whereas “unhealthy systems gravitate toward periodic and simplistic output” (Guastello, 2004). Chaos also plays a crucial role in brain dynamics, an area increasingly explored by neuropsychoanalysis (Grigsby and Stevens, 2000). We can also see examples from birds of what Deleuze and Guattari (2003a) call the territorializing effects of the familiar. Skarda and Freeman (1987, 161–195) see chaos “as an emergent property of intrinsically unstable neural masses.” Sole and Goodwin (2000, 138) explain how “chaotic (brain) dynamics (as shown by the observed strange attractors) represented the normal state when the animal was attentive” but that “these attractors underwent dramatic changes when some familiar odor was introduced” resulting in much more ordered neural fluctuation. The spatiotemporal pattern “exhibited a well-defined stable structure…characteristic for the specific odor” (Sole and Goodwin, 2000, 138).

**Figure 8:**
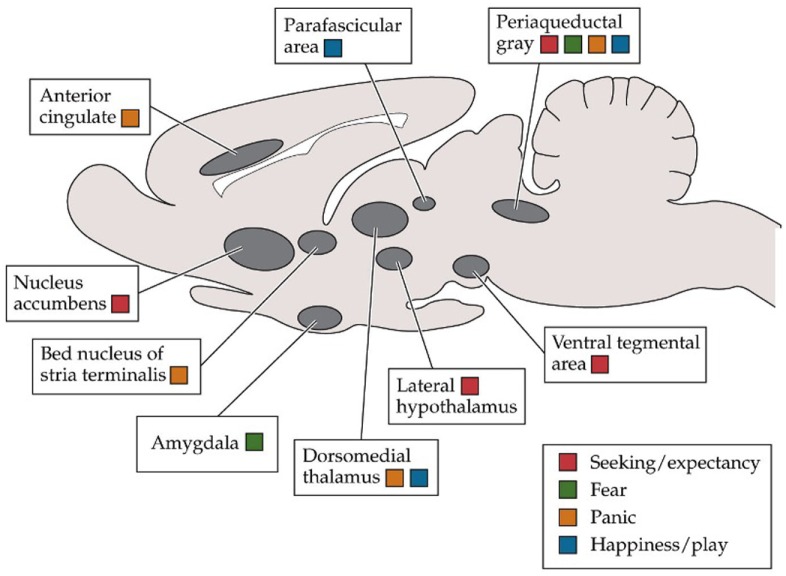
**Affect and attractor landscapes (Panksepp, 2005)**.

On the emotional level, Panksepp (2004), a pioneer of affective neuroscience and neuropsychoanalysis, argues that the basic emotion systems in the mammalian brain (see Figure 8) form *attractor landscapes* involving vast assemblages of neurons operating at far-from-equilibrium states. Paradoxically the non-linear processes of chaos give rise to stability by allowing the system to creatively adapt to environmental change, something increasingly urgent in our current crisis.

We can understand more fully the function of chaos through its border with more stable states, a regiom called the *edge of chaos*. Living systems attempt to balance themselves on the fractal border zone between stability and instability which provides maximum ecological flexibility, producing the *dissipative system* of life. Dissipative systems are open systems in constant reciprocal interaction with and adaptation to their environments and exist at *far-from-equilibrium* conditions where they can maintain themselves within a dynamically ordered structure. In ant colonies, for example, it has been noted that at a critical density “random individual activations become able to propagate through the whole colony, but the density is low enough to prevent activity from remaining a long time in the system” (Sole and Goodwin, 2000, 164). This is a fundamental challenge to long-held Western philosophical and scientific views on the relation between order and chaos as order arises from chaos in a specific scientific sense.

Kelso’s (1995, 26) work suggests that the brain “is a self-organizing, pattern-forming system that operates close to instability points, thereby allowing it to switch flexibly and spontaneously from one coherent state to another…by living near criticality, the brain is able to anticipate the future, not simply react to the present.” This can also be understood as a dynamic interplay between Deleuze and Guattari’s (2003a) deterritorialization/territorialization systems in constant flux. Camazine et al. (2001, 26) argues that “with such SO, environmental randomness can act as the ‘imagination of the system,’ the raw material from which structures arise” with fluctuations acting “as seeds from which patterns and structures are nucleated and grow.”

This principle can be seen as valid in all three of Guattari’s three ecologies of mind, society, and nature and has been applied to phenomena as far apart as organizational behavior (Dooley, 1997) and communication dynamics within families (Pincus, 2001). As Guastello (2004, 6) writes “The general principle is that the organism is a complex adaptive system, and that the turbulence or complexity in its behavior allows for the broadest range of adaptive responses.” With chaos, biology becomes no longer the “bedrock” on top of which separate psychological and social worlds form, because the brain is itself formed through non-linear interactions with the world, culture, and experience (Edelman, 2006).

In mental ecology, Marks-Tarlow (2004) reconfigures the psychoanalytic model of the self as a dynamical system, while Busch (2007, 429) describes pathological infantile attractors as “*black holes in psychological space*, sucking in everything in that comes near its orbit, remaining outside of awareness and thus unable to be modified by other structures.” Psychoanalysis can be understood as a coevolutionary system (Palombo, 1999) a “destabilization” of such attractors in psychic space, changing point, and periodic attractors to chaotic attractors. While most change is confined to the local level and absorbed by wider psychic defenses, as the system reaches *self-organized criticality* (Bak and Chen, 1991) the tiniest local shift can precipitate cascades of disorder through the entire system. Such models of dynamic change are also crucial for understanding the psychological and social shifts in human responses to ecological crisis.

In social ecology and group analysis, Stacey (2006), argues that Bion’s (1961) work group and basic assumption groups interact to create regions of stability and disintegration, with potentially creative fractal regions of bounded instability at the edge of chaos between them. Non-linear fractal geometry undermines any clear line between inside and outside, providing new ways to think about the individual and group in terms of multidimensional fractal border zones. Similarly, Jaques’ (1955) social phantasy systems can be understood as emerging through the SO of individual defenses, with global patterns feeding back to effect lower levels recursively. The nonlinear dynamical systems approach to intrapsychic, interpersonal and group psychodynamics also offers the rich possibility for modelling these complex systems using computer-based modelling techniques, something I call ‘artificial group psychodynamics’ (Dodds, 2009), allowing us to explore the parameters of our theories in a new form of empirical research.

For an example of a non-linear social phantasy ecosystem in climate change we can turn to Randall’s (2005) discussion of the non-active majority who project environmental concern onto activists functioning as containers for the split-off collective environmental superego. A non-linear social systems perspective lets us explore the affective feedback loops carried around the circuit with complex social and psychological effects, as projective and introjective identifications, splittings, and scapegoating, reverberate back and forth in new iterations as the system moves forward in time, as other individuals and groups get drawn in, either damping-out the mad oscillations (Bion, 1961) or getting swept up in non-linear amplification effects. Randall (2005, 176–177) suggests that as collective guilt becomes more shared, it can be “managed in more creative ways,” becoming “less persecutory and destructive” where projections are reduced and a larger non-psychotic space created for reparative action. This embodies a system of multistability, with complex movements between basins of attraction as internal objects and affects flow through the network, with major shifts between states, sometimes after long periods when the system seems stuck despite the best efforts to destabilize it by pushing it toward a bifurcation.

## Geophilosophy and The Future of the Three Ecologies

Here, we can draw on the evolutionary model of a “fitness landscape,” where each helpful mutation moves the species closer to a local adaptive peak, while the fitness landscape itself constantly morphs in new directions with the ebb and flow of evolutionary time. Just as a species can get “stuck” at a local optimum in the fitness landscape, a psychoanalytic patient is sometimes unable to make the temporary regression required to find new more creative ways of living (Palombo, 1999, 114). This is a novel account of “resistance” in psychotherapy and deadlocks in the wider culture concerning climate change, suggesting an inability to leave a local peak even when long-term consequences may be disastrous.

Using complexity theory, we can see our current period as showing disorder and instability in some areas, while seeming stuck and frozen in others. The first can feel frightening, the latter deadening and demoralizing (Marks-Tarlow, 2008). Periods of instability are “natural and necessary stages on the path toward greater SO” (Eidelson, 1997, 68) but with no guarantee that what emerges will be more adaptive. From a clinical point of view, it is interesting to note that there can be pathological, aberrant patterns in swarms (Camazine et al., 2001, 282). What this research shows is that in a highly complex and interconnected system, relatively small changes of one parameter can have unpredictable (and disastrous) effects on the whole. This has important implications for the effect of climate change on the social, psychological, climate, and ecological systems in Guattari’s (2000) three ecologies.

This can bring a complexity based approach to Jared Diamond’s (2006) research on the collapse of civilizations, and the important roles he uncovered for systemic social interconnectivity, environmental damage, and climate change. Crucially for us, many of these societies entered the period of collapse shortly after reaching to their apogee of power and wealth. The Anasazi Chaco society in North America, for example, built the tallest buildings in that continent prior to Chicago’s scrapers of the 1880s. Following centuries of development, their advanced society collapsed rapidly in one decade after reaching its peak, between the years 1110 and 1120. Climate change appears to have played an important part in the rise and fall of many previous civilizations, including ones as mighty as Rome (Buntgen et al., 2011). We do not yet know whether our own civilization will share the fate of many that have gone before, but we would do well to grasp the complex non-linear effects involved. Complexity theory lets us understand how psychological, natural, and social ecologies are organized, and how fragile they can be.

We still do not know why civilizations collapse. We need some new explorers able to penetrate the jungles of complexity and find the theories that will help us locate ourselves in our complex world…a fascinating but counterintuitive universe, a non-linear and unpredictable world operating by rules still to be discovered… They will tell us more than we can imagine about our brains and societies…Let us remember, when walking through the ruins of those great ancient cities…that they all had something in common: a long time ago, their citizens believed that those cities and those civilizations would last forever (Sole and Goodwin, 2000, 302).

Complexity and chaos theory shows how in certain conditions, even minor changes can produce dramatic effects. The task for change, whether in psychoanalysis or social systems, then becomes experimental, including the search for “lever points” to open up the possibilities of more radical transformation. As Deleuze and Guattari, 2003a, 161) write:

This is how it should be done: Lodge yourself on a stratum, experiment with the opportunities it offers, find an advantageous place on it, find potential movements of deterritorialization, possible lines of flight, experience them, produce flow conjunctions here and there, try out continuums of intensities segment by segment… It is through a meticulous relation with the strata that one succeeds in freeing lines of flight.

**Figure 9:**
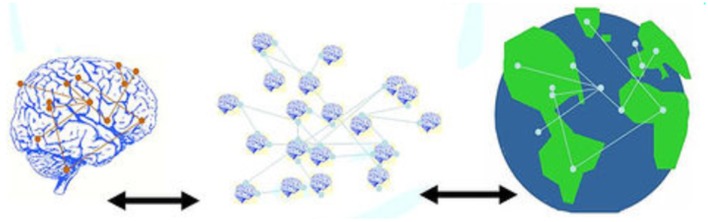
**Integrating different spatiotemporal scales**.

Deleuzo–Guattarian philosophy is one of becoming rather than being. Everything, from mountains to bodies to languages represent merely temporary hardenings or slowing down of the vast flow of becoming. DeLanda’s *assemblage theory* helps to situate this philosophy alongside the sciences of complexity and chaos. It studies how structures at all levels emerge through their interacting components. For DeLanda (2006, 119) this allows us to integrate insights from all spatiotemporal scales (Figure 9), forming “a chorus that does not harmonize its different components but interlocks them while respecting their heterogeneity.” Assemblage theory provides a way to approach all three of Guattari’s ecologies, in a dense heterarchy (Wilson and Holldobler, 1988) of connectivity, where a “territorial assemblage opens onto a social assemblage” which is also “connected to cosmic forces” and “pulsations of the earth” (Deleuze and Guattari, 2003a, 549).

Deleuze and Guattari create a vision of a world, according to DeLanda where “geology, biology, and linguistics are not seen as three separate spheres” but as “coexisting and interacting flows” where “one stratum can serve directly as a substratum for another.” As Deleuze and Guattari (2003a, 69) put it, “a semiotic fragment rubs shoulders with a chemical interaction, an electron crashes into a language.” Deleuze and Guattari follow Bateson toward an ecology of mind leading to new ways of understanding subjectivity, where fallacies in the ecology of ideas have direct and catastrophic results on the social and ecological registers such that “there is an ecology of bad ideas, just as there is an ecology of weeds” (Bateson, 2000, 492).

In our current ecological crisis we must face the possibility that achieving the necessary ecological flexibility to survive requires a fundamental re-examination of the basic coordinates of our lives. Like the patient stuck on a local optimum, unable or unwilling to cross the threshold to a more adaptive peak, entire species, and civilizations have in the past found themselves in dangerous dead ends; including those within the ecology of mind, ways of thinking, and being that become pathological if they fail to evolve along with the constantly shifting relations in the constitution of natural and social ecosystems. The contribution of psychoanalysis is to help us overcome such errors of thought through investigating their unconscious roots. Although there may be many defensive reasons to *not* know (Bion’s), we are starting to become conscious of the enormity of the danger which confronts us. The non-linearity and chaos of nature, and the forms of thinking required to sustain our relationship to it beyond the limited horizons of our experience, are both frightening and liberating. Yet, despite the anxiety, guilt, and terror that climate change forces us to face, this crisis can offer us an opportunity for a more open vision of ourselves, as subjects, as societies, and as a species, among the interconnected life systems of the Earth.

## Conflict of Interest Statement

The author declares that the research was conducted in the absence of any commercial or financial relationships that could be construed as a potential conflict of interest.
